# Gaze-based attention refocusing training in virtual reality for adult attention-deficit/hyperactivity disorder

**DOI:** 10.1186/s12888-023-04551-z

**Published:** 2023-01-26

**Authors:** Benjamin Selaskowski, Laura Marie Asché, Annika Wiebe, Kyra Kannen, Behrem Aslan, Thiago Morano Gerding, Dario Sanchez, Ulrich Ettinger, Markus Kölle, Silke Lux, Alexandra Philipsen, Niclas Braun

**Affiliations:** 1 Department of Psychiatry and Psychotherapy, University Hospital Bonn, Bonn, Germany; 2 Department of Psychology, University of Bonn, Bonn, Germany

**Keywords:** Virtual reality, Eye-tracking, ADHD, Adults, Attention training, Treatment, Therapy, Continuous performance task, Distractors, Self-regulation, Metacognition, EEG

## Abstract

**Background:**

Attention-deficit/hyperactivity disorder (ADHD) is characterized by substantial interindividual heterogeneity that challenges the systematic assessment and treatment. Considering mixed evidence from previous neurofeedback research, we present a novel feedback system that relies on gaze behavior to detect signs of inattention while performing a neuropsychological attention task in a virtual seminar room. More specifically, an audiovisual feedback was given whenever participants averted their gaze from the given task.

**Methods:**

Eighteen adults with ADHD and 18 healthy controls performed a continuous performance task (CPT) in virtual reality under three counterbalanced conditions in which either gaze-based feedback, sham feedback, or no feedback was provided. In all conditions, phases of high and low virtual distraction alternated. CPT errors and reaction times, proportions of gaze dwell times (e.g., task focus or distraction focus), saccade characteristics, EEG theta/beta ratios, head movements, and an experience sampling of ADHD symptoms were analyzed.

**Results:**

While patients can be discriminated well from healthy controls in that they showed more omission errors, higher reaction times, higher distraction-related dwell times, and more head movements, the feedback did not immediately improve task performance. It was also indicated that sham feedback was rather associated with an aggravation of symptoms in patients.

**Conclusions:**

Our findings demonstrate sufficient suitability and specificity for this holistic ADHD symptom assessment. Regarding the feedback, a single-session training was insufficient to achieve learning effects based on the proposed metacognitive strategies. Future longitudinal, multi-session trials should conclusively examine the therapeutic efficacy of gaze-based virtual reality attention training in ADHD.

**Trial registration:**

drks.de (identifier: DRKS00022370).

**Supplementary Information:**

The online version contains supplementary material available at 10.1186/s12888-023-04551-z.

## Introduction

With an estimated prevalence of 5% [1, 2], attention-deficit/hyperactivity disorder (ADHD) is the most common mental disorder in childhood. It is characterized by pervasive patterns of inattention, hyperactivity, and impulsivity that interfere with functioning [3]. In adults, the global prevalence is estimated at 2.58% [4] and symptoms of inattention are most pronounced [5]. Considerable psychosocial and health economic implications have been reported [6], particularly given the wide range and high rate of comorbidities associated with adult ADHD [7].

Stimulants are recommended as a first-line treatment in ADHD [8], whereas cognitive behavioral therapy is recommended in cases of low treatment benefit of medication or mild symptomatology. However, both treatment modalities have limitations. In adults, psychostimulants are reported to have mean response rates of only about 60% and are less effective and less well tolerated than in children and adolescents [9, 10]. Additionally, psychostimulant treatment responses have been found to depend on individual symptom profiles [11], might relate to genetic factors [12] and, while the risk of serious harm is considered low, some adverse effects have been reported [13, 14]. Psychotherapeutic interventions, in turn, are often restricted to cognitive behavioral approaches that improve coping mechanisms for ADHD symptoms and related difficulties [15], but address ADHD core symptoms less directly.

Moreover, ADHD is a disorder with substantial heterogeneity in clinical profiles, neurocognitive impairments and treatment responses [10, 16]. Consequently, a systematic review highlighted the need to integrate multilevel information for an effective exploration and treatment of the varying degrees of dysfunction and their respective symptom expression [17]. Given that treated patients with ADHD still report considerable burden of their symptoms in everyday life [18, 19], the development of more effective and specific therapeutic approaches is needed. Two relatively new treatment approaches, computerized cognitive training (CCT) and neurofeedback, thereby intend to directly target cognitive dysfunction associated with ADHD.

CCT aims to enhance various cognitive functions such as attention, reaction speed, or behavioral inhibition through repetition of computer-based cognitive tasks. Most of these trainings have been developed for children and adolescents with ADHD [20, 21] but almost none for adults [22]. In the few cognitive training programs available for adults with ADHD, effects were either not superior to an active control group or could not be generalized beyond performance enhancements within the specific training paradigm [23, 24]. This may be linked to the concept of CCTs often being developed to directly address neuropsychological symptoms, rather than to create awareness of environmental triggers and the specific consequences. Specifically, cognitive tasks for the treatment of ADHD often address the patients' difficulties in sustaining attention, but few focus on impairments in the metacognition of attentional functions or deficits in self-regulation, such as recognizing attentional misdirection and dealing with limited attentional capacity [25, 26].

Another underlying cause for the insufficient evidence for CCT in adult ADHD might derive from its abstract nature and lack of transferability to real-world situations, especially since the neuroscientific foundation of cognitive training appears well-grounded. Until now, CCT has been delivered almost exclusively on classic computer screens. Therefore, given the higher achievable degree of perceived realism and ecological validity, it would be of particular interest to offer CCT by using virtual reality (VR). VR is defined by the capability of a seemingly real user interaction with computer-generated simulations of an environment. A recent systematic review of neuropsychiatric rehabilitation based on cognitive training in fully immersive VR provided some promising evidence of its cognitive benefits [27].

In neurofeedback, in turn, a cognitive task is performed and real-time feedback on some specific aspects of one’s own, otherwise covert, brain activity is simultaneously received [28]. Repeated training is thought to result in an increase in the ability to modify one’s own brain signal and to thereby improve cognitive functioning. While various EEG-based [29], fMRI-based [30] and fNIRS-based [31] protocols have been developed for neurofeedback application, a modulation of the theta/beta ratio (TBR) in EEG is often the therapeutic objective in ADHD [32, 33].

In summary, however, although the general concept appears plausible, the existing evidence for neurofeedback is inconsistent, particularly with respect to long-term improvements in clinical outcomes of adult ADHD [29]. One of the contributing factors seems to be the unsolved issue of which brain signal should be considered for feedback and from which brain modality it should best be derived [28, 33]. In addition, various technological shortcomings such as the relatively low signal-to-noise ratio of EEG, the sensitivity of fMRI to motion artifacts and the rather low temporal resolution of fNIRS hinder the optimal implementation of neurofeedback. Moreover, while state-of-the-art neuroscientific research methodology provides a valid foundation for measures of attention [34] and ADHD symptoms have been differentiated for adulthood and characterized in detail [35], a gap remains for treating attention disorders at the clinical level. Therefore, interventions based on valid assessment methodology that explicitly aim at inattention behavior by initiating metacognitive learning processes, e.g., by improving attentional modulation, might be a promising approach to improve attentional dysfunction.

Conceivable advancements in the treatment of dysfunctional metacognition and self-regulation in ADHD might be achieved through eye-tracking, which features a high temporal resolution, a comparatively good signal-to-noise ratio and a user-friendly, unobtrusive application. While unlike in EEG, fMRI, or fNIRS, no measures of brain activity are captured directly, the objective quantification of eye movements is of particular value in the field of attention research [36, 37]. In this context, it is highly useful that humans are naturally inclined to pursue shifts in overt attention, i.e., physically directing their eyes to stimuli [38]. In ADHD, oculomotor inhibition, i.e., the ability to select relevant information and to reflexively suppress attending irrelevant or distracting stimuli, has been discussed as a potential biomarker of the disorder [39].

The assessment of eye movement behavior in ADHD is often conducted during the performance of a neuropsychological attention task, such as the continuous performance task (CPT). Here, participants must react upon infrequent target stimuli and withhold their responses to frequent non-target stimuli [40]. Adults with ADHD were found to gaze more at task-irrelevant areas than healthy individuals while performing a CPT during concurrent presentation of distractors [41]. While such distractibility is considered bottom-up driven, i.e., by environmental stimuli, mind wandering is a spontaneous, unintentional shift away from a task toward internal thoughts [42]. Spontaneous mind wandering is associated with increased functional impairments in ADHD [43] and has led to variations in eye movement behavior during attentional task performance in healthy individuals [44, 45]. Therefore, for the systematic detection and subsequent feedback provision that renders the awareness of both types of inattention, gaze tracking during a CPT may be a promising approach.

**Fig. 1:**
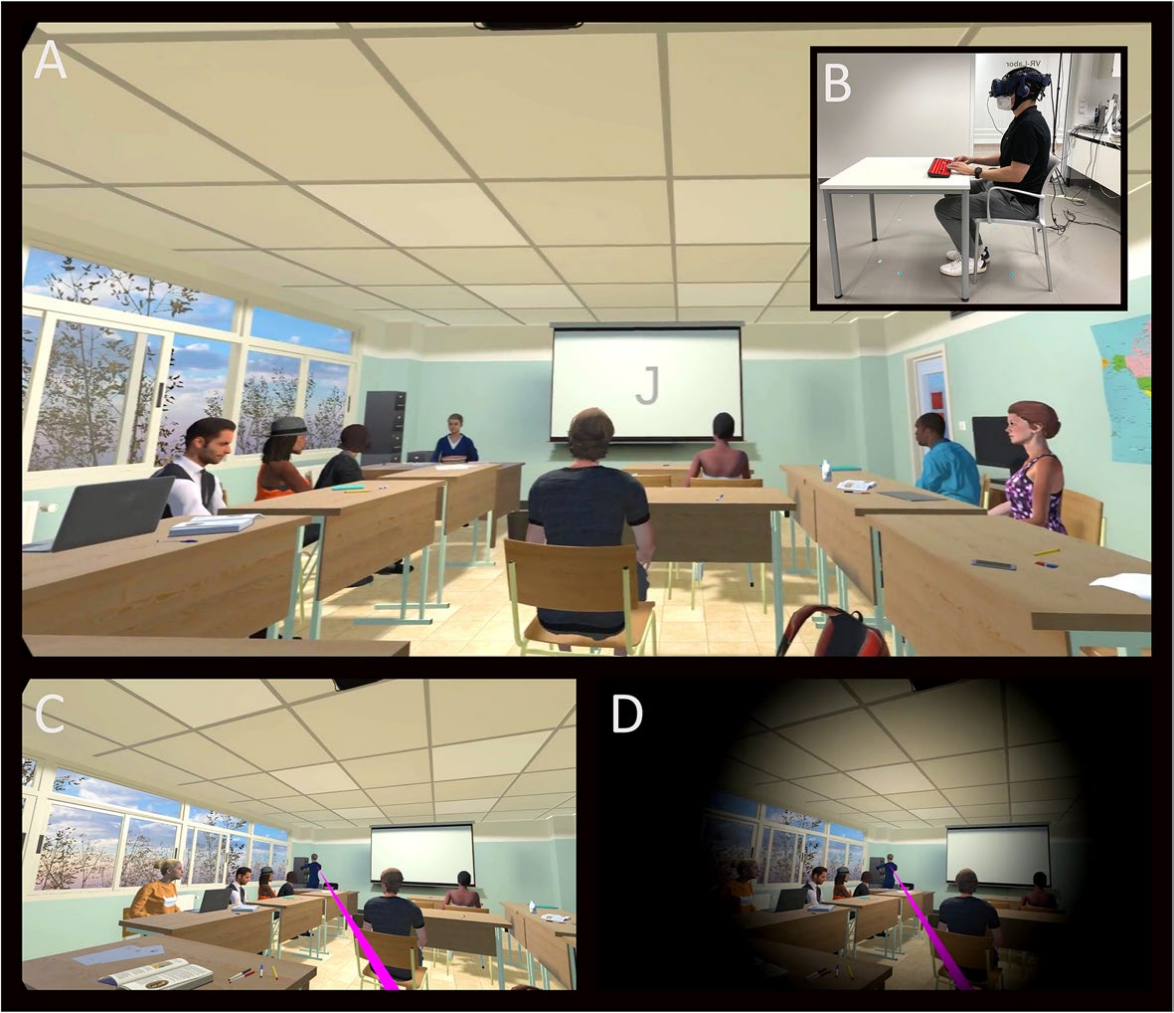
The virtual seminar room (VSR) into which the participants immersed via a head-mounted display. **A** First-person view of the virtual seminar room in which the continuous performance task is presented on the canvas at the front wall. **B** Real world side view of participant in the virtual reality lab. **C** One of the distractor events played during a distractor phase: avatar in the front is standing up and walking to a cabinet, thereby attracting the attention of the participant as indicated by the visualized pink gaze vector (not visible for study participants). **D** Gaze-based feedback provision. Whenever the participant’s gaze shifted away from the canvas for more than 2 s or the gaze was directed at a distractor for at least 0.5 s, audiovisual feedback was automatically played (combined black fade-in and sound effect). For a video presentation of this feedback, see Supplementary Material 1

Consequently, the aim of this study was the development and evaluation of what is, to our knowledge, the first gaze-based attention refocusing training in virtual reality (GART) for patients with ADHD. This system builds upon existing CCT and neurofeedback principles, but is intended to specifically target metacognitive and self-regulatory functions. More specifically, we applied our developed virtual seminar room (VSR) [46], and extended it with a gaze-based feedback system that intervenes each time a person stops attending a VSR-embedded CPT (see Fig. 1). To evaluate this GART, 18 adult patients with ADHD and 18 healthy controls (HC) performed our virtual CPT (including alternating phases of additional distraction) in three counterbalanced feedback conditions: a *real feedback* condition, in which audiovisual feedback was given as soon as participants averted their gaze from the task-relevant canvas; a *sham feedback* condition, in which the feedback was triggered with a quasi-random delay; and a *no feedback* condition in which no feedback was given at all. A multimodal offline evaluation of CPT performance measures, psychophysiological measures (eye movements, EEG, head actigraphy) and subjective ratings was conducted.

## Methods

### Participants

**Fig. 2:**
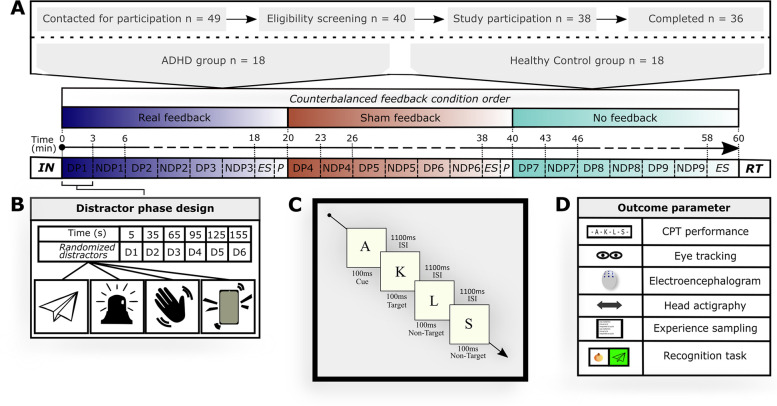
Participant flow and experimental design. **A** 36 participants underwent all three feedback conditions in counterbalanced order on experiment Day 2. First, instructions were shown and a short continuous performance task (CPT) trial block was run. Then, the task block started, combined with either the real feedback, sham feedback, or no feedback. Following each 18-min CPT block, participants underwent experience sampling (ES) and a short break (P). Within each feedback block, time phases with distracting events (DP) and phases without distracting events (NDP) were alternated in three-minute cycles. At the end of the experiment, the VRSQ was completed and a recognition task (RT) regarding presented distractors was conducted. **B** Distractor phase design. Audio, visual, or audiovisual distractors were presented every 30 s during DP. **C** Implementation of the CPT. The CPT was presented on a canvas with a stimulus interval of 100 ms and an interstimulus interval of 1100 ms. **D** Outcome parameters of the study. *Abbreviations:* ADHD: Attention-deficit/hyperactivity disorder, ES: Experience sampling, D: Distractor, DP: Distractor phase, HC: Healthy control, ISI: Interstimulus interval, NDP: Non-distractor phase, P: Pause, RT: Recognition task, VRSQ: Virtual Reality Sickness Questionnaire

The study was advertised via the adult ADHD specialist outpatient clinic of the Department of Psychiatry and Psychotherapy of the University Hospital Bonn and via publicly accessible media. Of 40 participants who entered the study between February 2021 and August 2021, 36 completed the participation (for the participant flow, see Fig. 2). To be eligible, participants had to be between 18 and 65 years of age, have normal or corrected-to-normal vision, adequately understand the study content and language, not be pregnant, not have epilepsy, not have oculomotor atypicalities, and not have rashes on the scalp. Moreover, all participants assigned to the ADHD group had to meet the DSM-5 diagnostic criteria for ADHD as assessed with the revised, German version of the Clinical Interview for the Integrated Diagnosis of ADHD in Adulthood (IDA-R) [3, 47]. Additionally, they had to be free of a schizophrenia spectrum disorder, severe affective disorder, antisocial personality disorder, or moderate-to-severe substance abuse. Also, participants had to discontinue taking their ADHD medication 48 h before the experiment. Healthy participants, in turn, were ineligible if they had a psychiatric diagnosis as mentioned above or a diagnosis of ADHD. Therefore, before study participation, all potential participants were screened with the Brief Diagnostic Interview for Mental Disorders (Mini-Dips-OA, German version) [48] and the Assessment of DSM-IV Personality Disorders (ADP-IV, German version) [49].

The study was conducted in accordance with the Helsinki Declaration as revised in 2013, and approved by the local medical ethics committee of the University of Bonn (protocol number: 297/20). A required sample size of 36 participants was revealed by an a priori sample size calculation in G*Power [50], based on an alpha error probability of 0.05, a power of 0.9 and a moderate effect size. Written informed consent was obtained from all participants. Information that could identify participants is not published. For compensation, participants had the opportunity to enter a draw (2 × 50 €). The trial was preregistered in the German WHO primary registry DRKS on 01–12-2020 (identifier: DRKS00022370).

### Study design

The study was designed as a two-armed controlled trial, in which two groups (patients with ADHD, HC) received three feedback conditions in counterbalanced order: real feedback, sham feedback, and no feedback. The real feedback condition thereby served as the main intervention of interest, during which audiovisual feedback was triggered as soon as an eye-tracked gaze behavior was registered that indicated a loss of task focus (details below). The sham feedback and no feedback conditions, in turn, served as control conditions. Whereas in the sham feedback condition, the same type of audiovisual feedback was provided as in the real feedback condition, except that here the feedback was provided with a quasi-random delay (20—30 s) after inattention registration, the no feedback condition provided no feedback at all. Otherwise, all three conditions were identically structured: Participants were immersed into the VSR, i.e., a virtual testing environment of high ecological validity for the multimodal assessment of ADHD-associated symptoms, and performed a CPT while distracting events occurred. Participants were blind to which CPT block represented which condition, but were informed that feedback could appear at any time and in any condition.

Study participation was scheduled over two days: Day 1 served for the eligibility testing and clinical characterization of our participants and was conducted online as a result of COVID-19 restrictions for some participants. Day 2 included the experiment and occurred at the virtual reality laboratory of the Department of Psychiatry and Psychotherapy of the University Hospital Bonn. The total duration was approximately 1.5 h and 2.5 h for Day 1 and 2, respectively.

### Clinical characterization

ADHD symptoms were evaluated based on both the observer-rated clinical interview IDA-R [47] and the self-rating behavior questionnaire ADHS-SB [51]. Moreover, the World Health Organization Quality Of Life questionnaire (WHOQOL) [52] and the Depression, Anxiety and Stress Scales (DASS) [53] were completed for further clinical characterization. Demographic data were collected with a lab-internal questionnaire.

### Experimental procedure and virtual environment

The experimental procedure on Day 2 was as follows: First, participants were prepared for the EEG recordings, before they were seated at a 1 × 1 m table with a keyboard in front of them. Next, the head-mounted display (HMD) was placed on the participants' heads. The HMD used was the HTC Vive Pro Eye (HTC Corporation, Taoyuan City, Taiwan), which has 1440 × 1600 pixels per eye image resolution, a 90 Hz screen refresh rate, a 110-degree field of view and an embedded eye-tracking system. Immersed into the VSR, participants found themselves seated at a virtual table from where they could follow the VSR scenery from a first-person perspective (cf. Fig. 1). Besides the canvas, which was located at the front of the VSR and on which the CPT was presented, typical seminar room equipment and animated study mates were included. The VSR has been self-assembled by our lab using Unity 3D 2019.1.10f1 (Unity Technologies, San Francisco, CA, USA) and C# based on pre-existing assets (e.g., 3D Everything's School Classroom which is available in the Unity Asset Store). Its complete functionality and validation has previously been described in detail [46]. After the participants had briefly accustomed to the virtual environment, a short calibration sequence for the eye-tracking system followed, before a first trial run of the ensuing CPT task was conducted. Next, the three feedback conditions were run, with two-minute breaks and recalibrations of the eye-tracker between each condition. All three conditions consisted of performing a CPT for 18 min (cf. section continuous performance task), while additional distractor events occurred (cf. section implementation of distracting events) and, if applicable, audiovisual feedback was given. Each condition ended with an experience sampling, in which the participants were briefly surveyed about their subjective experiences via a VR-embedded survey tool. In addition, after all CPT blocks were completed, the Virtual Reality Sickness Questionnaire (VRSQ) [54] was presented, before participants removed the HMD. In total, participants remained in the virtual environment for about one hour. Finally, participants completed a recognition test regarding perceived distractor stimuli during the virtual experiment via a desktop screen.

### Continuous performance task

The CPT was directly presented on a canvas at the front wall of the VSR (cf. Fig. 1). Specifically, a sequence of single letters was presented centrally and iteratively on the canvas, with a stimulus duration of 100 ms and an interstimulus interval of 1100 ms, resulting in 900 trials per block. The task required pressing a key as quickly as possible when a "K" was shown after an "A", while withholding the response for any other sequence of letters. Compared to our previous VSR study [46], in which we found a ceiling effect (i.e. a very low error rate), a faster stimulus sequence was applied by decreasing the interstimulus interval by 800 ms. In each CPT block (i.e. each condition), 30% target sequences and 70% non-target sequences were presented. Of the latter, 50% were pseudo-targets containing only one of the two target letters. For analysis, reaction times (in ms) of all responses, commission errors (as an estimation of impulsivity) and omission errors (as an estimation of inattention) were derived.

### Implementation of distracting events

Each CPT block (i.e., condition) was further divided into alternating distractor phases (DP) and non-distractor phases (NDP), with each of these phases lasting three minutes. Whether a CPT block started with a DP or NDP was counterbalanced across participants. During an NDP, the seminar room was presented unchanged. During each DP, a total of six different visual, auditory, and audiovisual distractors were randomly selected (from a pool of 18 visual, 18 auditory, 18 combined audiovisual distractors) and presented in intervals of 30 s. The distractors represented events with high everyday relevance, such as a smartphone ringing or birds flying past the window and were widely balanced (28:26) in terms of their content reflecting a social (e.g., a person entering the room) or non-social (e.g., a passing fire truck) context.

### Eye-tracking recording

Eye movements were recorded with a sampling frequency of ~ 50 Hz via the infrared-based Tobii eye-tracker (Tobii Technology, Stockholm, Sweden) built into the HMD. The eye-tracker has an accuracy estimation of 0.5°—1.1° and allows the additional wearing of glasses, which was required in 39% of patients and 11% of healthy participants. Participants were asked not to wear any eye makeup. Eye-tracking data were recorded by a combination of three different software packages: SRanipal SDK version 1.3.1.1 (HTC Corporation, Taoyuan, Taiwan), Tobii XR SDK version 1.16.36.0 (Tobii Technology, Stockholm, Sweden), and Lab Streaming Layer (LSL; https://github.com/sccn/labstreaminglayer). SRanipal SDK provided access to the raw eye-tracking data within Unity. Tobii XR SDK was used to track the participant’s momentary gaze focus on specific virtual objects within Unity. Technically, this tracking was realized by the SDK’s *IGazeFocusable* interface that builds upon Unity’s collider system and allows to register whenever a specified collider (3D object) is hit by a raycast representing the participant’s gaze direction. Using this functionality, three different eye gaze states were defined and tracked:

- *Task focus:* The participant's gaze was fixed on the canvas on which the CPT was presented.
- *Distractor focus:* The participant's gaze was shifted to the collider of a 3D object, which was played as an animated distractor. In the case of purely auditory distractors, generous colliders were placed in the area where the sound source was located in the 3D environment.
- *Gaze wandering:* The participant’s gaze was neither directed to the canvas nor to a distractor-related 3D object, but to somewhere else in the virtual space. Gaze wandering here is intended to provide an eye movement-based estimate of mind wandering.

For each recorded time stamp, only one of the three possible gaze direction states (excluding blinks) was thereby possible at a time. Finally, LSL was used to save the eye-tracking data along with the other data collected.

### Implementation of the gaze-based online feedback

As stated, during both the real feedback and sham feedback conditions, audiovisual feedback was triggered whenever gaze locations indicated a loss of task focus. A loss of task focus was assumed as soon as a participant did not look at the canvas for more than 2 s or as soon as a participant gazed at a distractor for more than 0.5 s. In the real feedback condition, this resulted in an immediate provision of audiovisual feedback. In the sham feedback condition, an initial delay of 20—30 s was implemented before feedback initiation to ensure a similar frequency compared to the real feedback. The audiovisual feedback itself consisted of a 0.5 s black fade-in effect to a maximum of approximately 35% of the screen size combined with a chime-like sound effect (for a video presentation, see Supplementary Material 1). It was automatically stopped as soon as either the gaze was redirected to the canvas or 2 s passed. In addition, following feedback, there was a refractory period of 5 s during which no further feedback could be played to prevent over-extensive initiation of feedback.

### Eye-tracking offline analysis

Eye-tracking offline analyses were conducted in Matlab 2021b (The MathWorks Inc., Natick, MA, USA). Detection of saccades and fixations was based on a custom Matlab script that implemented an adaptive data-driven algorithm for velocity-based detection (for details, see [55]). Specifically, the three-dimensional gaze coordinates of each eye were used to calculate sample-to-sample velocities and accelerations [56]. A second order Savitzky-Golay finite impulse response filter was applied for data smoothing [57]. Invalid data as indicated by the SRanipal validity score were discarded from analysis. Interpolation across gaps of 75 ms maximum duration was performed linearly. Loss of data from one side was compensated using valid data from the other eye, and subsequently the data from both eyes were averaged. Implicitly detected fixations with a duration of less than 60 ms were discarded and fixations were merged on the basis of inter-fixation intervals of maximum 40 ms. Mean data loss was 2.75% (SD = 1.98%) per participant. For analysis, the average number of saccades and average saccade durations (in ms) were derived for each condition and phase.

The analysis of the gaze direction behavior, in turn, focused on the three gaze direction states, which were already online determined in Unity during the experiment and tracked by LSL. For statistical analyses, the following dwell times were separately derived for each group and each feedback condition and, additionally, a composite distractibility score was calculated:

$$Distractibility\;score=\frac{Time\;of\;distractor\;focus\;\left(in\;\%\right)\;+\;Time\;of\;gaze\;wandering\;(in\;\%)}{Time\;of\;task\;focus\;(in\;\%)}$$

A high distractibility score thereby indicates a high level of distraction.

### EEG recording and analysis

The EEG was gathered via a wireless EEG system (Smarting®, mBrainTrain®, Belgrade, Serbia) and electrodes were placed by means of an EEG cap (EASYCAP, Herrsching, Germany) according to the 10–20 system and included 24 Ag/AgCl sintered ring electrodes: Fp1, Fp2, AFz, F3, Fz, F4, T7, C3, Cz, C4, T8, CPz, P7, P3, Pz, P4, P8, POz, O1, O2, M1, and M2, with the ground electrode (DRL) at FPz and the reference electrode (CMS) at FCz. With impedances kept < 10 kΩ, EEG data was digitized via LSL at a 500 Hz sampling rate and a 24-bit step-size resolution.

For the offline analysis, Matlab 2021b and EEGLAB 2021.0 [58] were used. First, the EEG datasets were temporally filtered between 0 and 35 Hz, detrended, and subsequently screened for noise in EEG channels. In each of 4 datasets, one noisy EEG channel was identified and replaced via spherical interpolation. Next, for calculating an independent component analysis (ICA), the continuous EEG data was epoched into 2 s time windows and non-stereotypic artifacts were removed using built-in EEGLAB functions. After that, an ICA was computed and components containing stereotypical artifacts such as ocular, cardiac, or muscle activity, were visually identified, backprojected to the continuous EEG data, and then rejected. The visual inspection of the components was thereby conducted by a trained EEG researcher and based on built-in functions of EEGLAB and focused on the components scalp topographies, spectral characteristics and time courses. The ICA-corrected continuous EEG datasets were cut into six separate subsets (either all DP or NDP within one feedback condition). Subsequently, every subset was epoched into as many non-overlapping five-seconds segments as possible, these segments were baseline corrected and all segments containing nonstereotyped artifacts were rejected. A continuous wavelet transformation was calculated on each retained segment for channel Fz. The time resolution amounted to 4 ms and the frequency range ranged from 0.1 to 35.0 Hz in 85 steps on a log scale. Finally, the average theta (4—7 Hz) and beta (13—30 Hz) power across segments was calculated between 0.5 and 4.5 s and the TBR was derived by dividing the theta power values by the beta power values.

### Head actigraphy recording and analysis

Head movement as a measure of actigraphy was obtained from built-in positional tracking of the HTC Vive system. The Euclidean 3D coordinates were recorded via LSL with a ~ 90 Hz sampling rate. For offline analysis in Matlab 2021b, the raw data was first downsampled to ~ 10 Hz and then the Euclidean distances between each consecutive 3D position of the HMD were computed. Finally, the mean distances of head position shifts were obtained.

### Experience sampling

After each feedback condition, a gesture-controlled user interface was provided to assess the participant’s momentary ADHD core symptoms. The user interface showed up as a semi-transparent overlay directly within the VSR and evaluated the participant’s symptoms of inattention, hyperactivity, and impulsivity on a 7-point Likert scale from -3 (no symptoms) to 3 (serious symptoms). Also, satisfaction with the GART and cybersickness via the VRSQ were inquired via this user interface.

### Recognition task

After completion of the experiment, a recognition task was administered in which 60 visual or auditory distractors were shown. Of these, 50% represented actual distractors played during the experiment and 50% represented distractors that were unplayed. Upon each presented distractor, participants had to decide whether this distractor was encountered during the experiment, or not. The recognition accuracy, i.e., the proportion of correct responses out of all possible correct responses, was derived for analysis.

### Statistical analyses

Complete data sets were available for all variables except the recognition task, which was not completed by two participants. The corresponding analysis of the recognition task was based on the remaining complete data sets.

With regard to ANOVA assumptions, visual inspection of Q-Q plots and histograms indicated non-normally distributed data in some cases. However, no serious violations were detected, and given the robustness of ANOVAs to non-normality [59], analyses were continued as planned. Sphericity violations were adjusted with the Huynh field correction for *ε* > 0.75 and Greenhouse–Geisser corrections in the remaining cases (see Supplementary Material 2).

To investigate potential differences between groups, feedback conditions, and phases, separate 2 × 3 × 2 mixed ANOVAs with the between-subjects factor Group (ADHD vs. HC) and the within-subject factors Feedback Condition (real feedback vs. sham feedback vs. no feedback) and Phase (DP vs. NDP) were carried out on commission errors, omission errors, reaction times, saccade durations, number of saccades, TBR values, and head movements. Moreover, 2 × 3 mixed ANOVAs (Group × Feedback condition) were conducted on gaze dwell time percentages (task focus, distractor focus, and gaze wandering), the composite distractibility score, and on the separate ADHD core symptom outcome scores of the experience sampling (inattention, hyperactivity, and impulsivity). Post-hoc comparisons were based on Bonferroni-adjusted t-tests. Independent samples t-tests were conducted to assess group differences with respect to VR-related cybersickness and satisfaction with the GART. Additionally, one-sample t-tests were carried out against "0" (moderate cybersickness/satisfaction) to determine differences from neutral responses. The accuracies of the recognition task were compared between both groups by using an independent samples t-test.

Finally, for an investigation of potential associations between measures, Pearson and Spearman's rank correlations with Benjamini–Hochberg corrected *p*-values were calculated separately for each group and feedback condition for several outcome parameters [60]. These included all previously described CPT and eye movement parameters, EEG theta power and beta power, head movements, the number of feedback triggered (except for the no feedback condition), and the ADHD total symptom scores as measured by experience sampling, the IDA-R, and the ADHS-SB. Age and education were the only demographic parameters evaluated.

All statistical tests were performed two-sided with a significance level of *α* = 0.05. Due to the exploratory nature of this study, which, to our knowledge, is the first to implement such simultaneous recording of multimodal physiological and behavioral data streams in VR, and which is intended to act hypothesis-generating for future confirmatory trials, unadjusted p-values are presented (except for the correlation analyses) with respect to multiple testing [61, 62]. Analyses were performed in SPSS 21.0 (IBM Corp., Armonck, NY, USA), except for the correlation analyses, which were performed in Matlab 2021b (The MathWorks Inc., Natick, MA, USA) and R software 3.6.1 [63] and visualized by means of the Corrplot package for R version 0.84 [64].

## Results

The detailed results of each ANOVA are summarized in Supplementary Material 2 (Supplementary Tables 1—5).

### Sample characteristics

Overall, 18 adult outpatients with ADHD (6 females) and 18 HC (7 females) participated in the present study. All of them were recruited in Germany and identified as of White European ethnicity. Detailed sample characteristics are presented in Table 1.

**Table 1:** Demographic and clinical characteristics

	**No. (%)**		***p*-value**^**c**^
**Characteristic**	**ADHD (*n* = 18)**	**HC (*n* = 18)**	**Group comparisons**
Age, y (SD)	36.1 (10.7)	25.9 (3.1)	.001
Female	6 (33.3)	7 (38.9)	.73
Right handed	17 (94.4)	15 (83.3)	.60
Education			.027
≤ Intermediate certificate	6 (33.3)	0	
Higher education entrance qualifications	6 (33.3)	9 (50.0)	
Higher education degrees	6 (33.3)	9 (50.0)	
Full- or part-time employment	9 (50.0)	15 (83.3)	.075
Married or living with a partner	8 (44.4)	12 (66.7)	.32
IDA-R ADHD symptom severity, mean (SD)	33.6 (7.3)	7.4 (5.5)	< .001
Inattention	18.8 (3.1)	4.8 (3.6)	< .001
Hyperactivity	7.8 (3.6)	1.3 (1.8)	< .001
Impulsivity	6.9 (2.6)	1.2 (1.8)	< .001
ADHD presentations			
Predominantly inattentive	7 (38.9)		
Predominantly hyperactive-impulsive	0		
Combined presentation	11 (61.1)		
Current psychopharmacological treatments			
Methylphenidate/Amphetamine	11 (61.1)	0	< .001
Antidepressant	2 (11.1)	1 (5.6)	.55
Current comorbid psychiatric disorders^a^			
Affective disorders	0	0	
Anxiety disorders	8 (44.4)	2 (11.1)	.060
Other disorders	2 (11.1)	0	.49
Comorbid psychiatric disorders in remission^a^			
Affective disorders	12 (66.7)	2 (11.1)	.002
Anxiety disorders	4 (22.2)	0	.10
Other disorders	1 (5.6)	1 (5.6)	1.00
DASS depression score, mean (SD)	10.1 (1.7)	8.8 (4.2)	.24
DASS anxiety score, mean (SD)	10.6 (2.7)	8.6 (2.5)	.030
WHOQOL quality of life total score, mean (SD)^b^	59.6 (11.8)	80.6 (13.6)	< .001

*DASS* Depression Anxiety Stress Scales, *IDA-R* Integrated Diagnosis of ADHD in adulthood—Revised, *WHOQOL* World Health Organization Quality Of Life

^a^Assessed on the diagnostic short interview for mental disorders [48]. Note that current severe affective disorders were an exclusion criterion for study participation

^b^Total score calculated as the mean of the four subscales, transformed to range 0–100, with higher values indicating a higher subjective quality of life

^c^Results of independent-samples t-tests, respectively chi-squared tests, are reported

### CPT performance

**Fig. 3:**
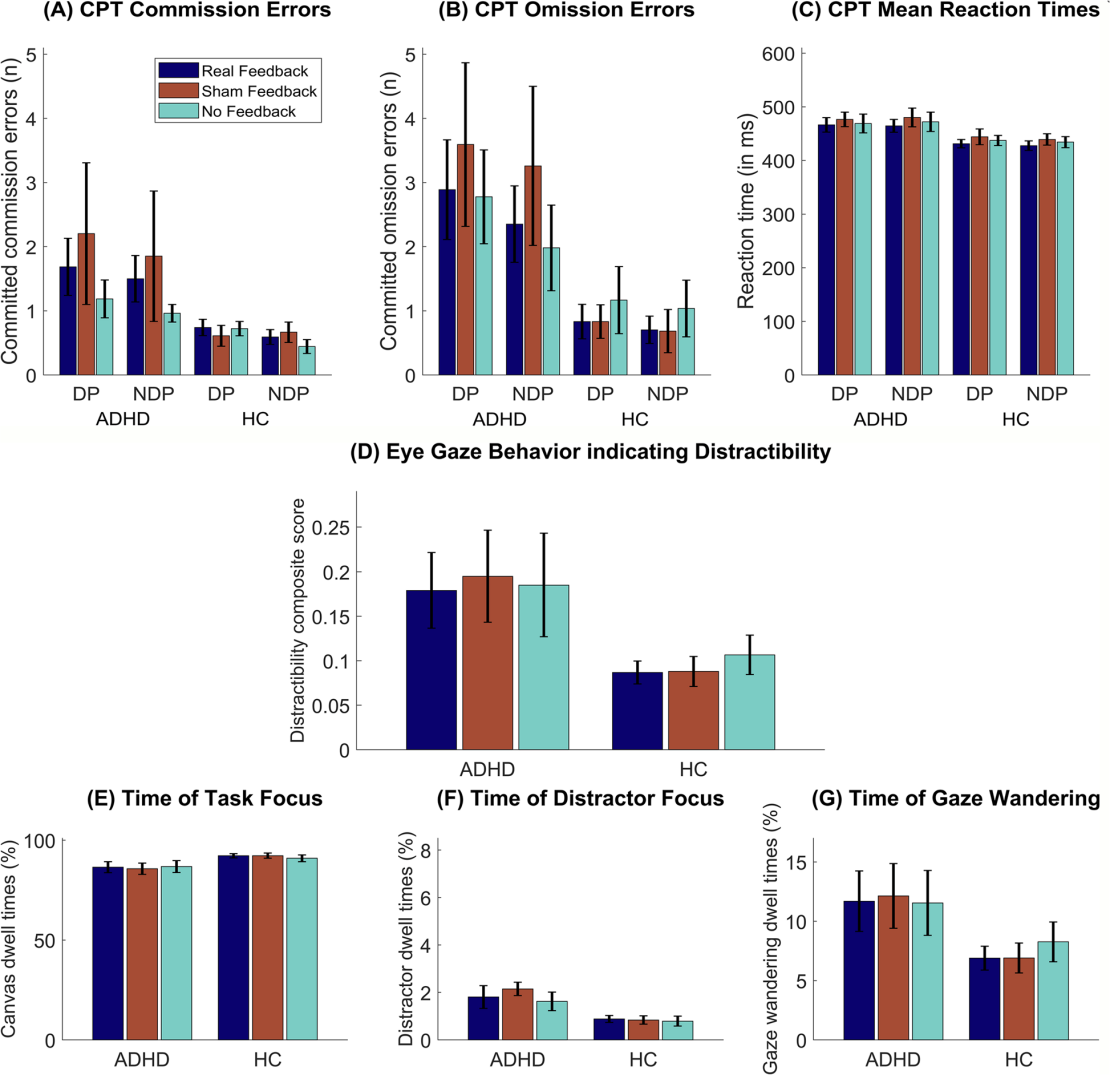
Results of the continuous performance task (**A**—**C**) and gaze behavior analysis (**D**—**G**)*.* The number of (**A**) commission errors, (**B**) omission errors and (**C**) mean reaction times are depicted for each feedback condition and both distractor phase types. **D** A composite distractibility score of the participants’ gaze behavior is depicted. The score reflects the sum of (**F**) the time spent gazing on distractors and (**G**) gaze wandering, divided by (**E**) the amount of time participants were looking onto the canvas on which the continuous performance task was presented. **E** to **G** show relative times for each of the three derived gaze parameters. Bars represent feedback conditions and are grouped by patients with ADHD and HC. Error bars indicate the SEM. *Abbreviations:* ADHD: Attention-deficit/hyperactivity disorder, CPT: Continuous performance task, DP: Distractor phase, HC: Healthy control, NDP: Non-distractor phase

The results of the behavioral CPT performance are depicted in Fig. 3 (A—C). No significant main effect of Feedback Condition or interactions between Phase, Feedback Condition and Group were detected for reaction times, omission errors or commission errors.

For omission errors, a significant main effect of Phase (*F*(1,34) = 9.35, *p* = 0.004, η*p*^2^ = 0.22) was found, in that significantly more omission errors were made during DP (*M* = 2.02; 95% CI [1.11, 2.92]) compared to NDP (*M* = 1.67; 95% CI [0.90, 2.44]). Likewise, at least descriptively (*F*(1,34) = 3.74, *p* = 0.061, η*p*^2^ = 0.10) more commission errors were observed during DP (*M* = 1.19, 95% CI [0.58, 1.80]) than NDP (*M* = 1.00, 95% CI [0.51, 1.49]).

Patients with ADHD and HC differed in omission errors (*F*(1,34) = 5.57, *p* = 0.024, η*p*^2^ = 0.14) and reaction times (F(1,34) = 4.37, *p* = 0.044, η*p*^2^ = 0.11). Across phases and feedback conditions, the ADHD group committed more omission errors (*M*_OE_ = 2.81, 95% CI [1.63, 3.99]) and had slower reaction times (*M*_RT_ = 471.46 ms, 95% CI [446.85, 496.07]) than the HC group (*M*_OE_ = 0.88, 95% CI [-0.30, 2.05]; *M*_RT_ = 435.64 ms; 95% CI [411.03, 460.26]).

### Gaze behavior

To evaluate the participants' gaze behavior during CPT performance, four gaze direction parameters were analyzed (cf. Fig. 3D - G): time of task focus, time of distractor focus, time of gaze wandering, and a composite distractibility score. For none of the four gaze direction parameters, a significant main effect of Feedback Condition or an interaction between Feedback Condition and Group was shown.

Instead, a significant group difference was found regarding the time of distractor focus (*F*(1,34) = 9.40, *p* = 0.004, η*p*^2^ = 0.22), in that patients with ADHD spent more time (M = 1.86%; 95% CI [1.38%, 2.34%]) gazing at distractors than HC (*M* = 0.84%; 95% CI [0.36%, 1.32%]). Comparing the time of attending the canvas between the ADHD group (*M* = 86.35%; 95% CI [82.14%, 90.56%]) and HC (*M* = 91.81%; 95% CI [87.60%, 96.01%]), healthy individuals showed only descriptively a higher percentage (*F*(1,34) = 3.48, *p* = 0.071, η*p*^2^ = 0.09). In line with these indications, there was also a trend for a higher distractibility composite score in patients compared with HC (*F*(1,34) = 3.68, *p* = 0.064, η*p*^2^ = 0.10).

### Saccade behavior

For the average duration and the number of saccades, the ANOVAs revealed no interactions, but showed longer (*F*(1,34) = 11.73, *p* = 0.002, η*p*^2^ = 0.26) and a higher number of saccades (*F*(1,34) = 13.87, *p* = 0.001, η*p*^2^ = 0.29) during DP than NDP. In addition, we found a higher number of saccades (*F*(1,34) = 4.90, *p* = 0.034, η*p*^2^ = 0.13) but only descriptively longer saccade durations (*F*(1,34) = 3.90, *p* = 0.057, η*p*^2^ = 0.10) in ADHD than in HC.

### EEG 

**Fig. 4:**
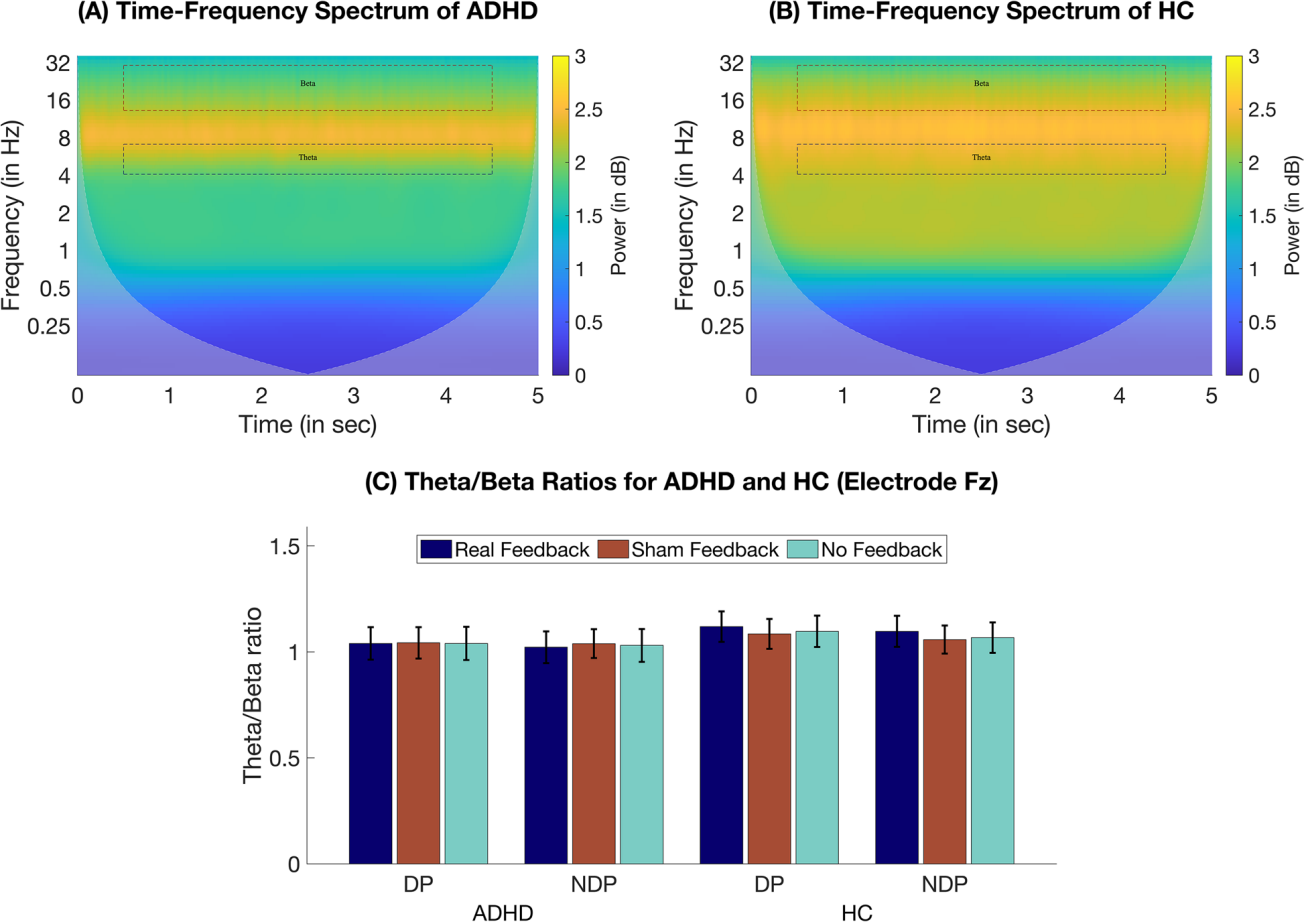
EEG wavelet analysis*.* Time–Frequency spectra of the wavelet analysis for (**A**) patients with ADHD and (**B**) HC across feedback conditions and phase types at electrode Fz. Dashed squares indicate analyzed time windows of interest (0.5—4.5 s) and frequency ranges of interest (theta [4 -7 Hz], beta [13—30 Hz]). **C** Comparison of the theta/beta ratio power for each group and between distractor phases and feedback conditions. Error bars indicate the SEM. *Abbreviations:* ADHD: Attention-deficit/hyperactivity disorder, DP: Distractor phase, HC: Healthy control, NDP: Non-distractor phase

EEG analyses focused on spectral differences concerning the participants' TBR. Time–frequency power spectra of the conducted wavelet analyses are presented in Fig. 4A and B, whereas the TBRs are depicted Fig. 4C. We found no significant main or interaction effects for Feedback Condition or Group, but a main effect of Phase (*F*(1,34) = 18.02, *p* < 0.001, η*p*^2^ = 0.35), in that the TBR was higher during DP (*M* = 1.07; 95% CI [0.97, 1.18]) than NDP (*M* = 1.05; 95% CI [0.95, 1.16]).

### Actigraphy 

Actigraphy analyses focused on differences in head movements. While the ANOVA indicated a significant main effect of Feedback Condition (*F*(2,68) = 3.58, *p* = 0.033, η*p*^2^ = 0.10), Bonferroni-adjusted post-hoc comparisons only yielded a trend (*p* = 0.053) toward more head movements during sham feedback compared to no feedback (*M*_*Diff*_ = 0.20; 95% CI [-0.002, 0.40]). We further found a significant main effect of Group (*F*(1,34) = 16.06, *p* < 0.001, η*p*^2^ = 0.32), in that patients with ADHD exhibited more head movements (*M* = 1.75; 95% CI [1.41, 2.09]) than HC (*M* = 0.80; 95% CI [0.46, 1.14]).

### Experience sampling 

To determine the subjective experience of momentary ADHD symptomatology, a short experience sampling was conducted after each CPT block, in which the participants rated their levels of inattention, impulsivity and hyperactivity. For none of the three parameters, any significant main effect of Feedback Condition or interaction effect emerged. Nonetheless, we found significant group differences for symptoms of inattention (*F*(1,34) = 19.57, *p* < 0.001, η*p*^2^ = 0.37), hyperactivity (*F*(1,34) = 16.96, *p* < 0.001, η*p*^2^ = 0.33), and impulsivity (*F*(1,34) = 8.76, *p* = 0.006, η*p*^2^ = 0.21), in that for all three ADHD symptoms higher ratings were observed in patients with ADHD than in HC.

The participants’ VR-related cybersickness was rated significantly lower than “0” (moderate sickness) by means of the VRSQ (*t*(35) = -2.76, *p* = 0.009, *d* = -0.46). Groups did not differ significantly (*t*(34) = 1.45, *p* = 0.156, *d* = 0.49) and, on average, scores of -0.25 (95% CI [-0.88, 0.38]) in the ADHD group and -0.78 (95% CI [-1.25, -0.32]) in the HC group were obtained. In line with this, no adverse events were reported with respect to the VR experiment.

Additionally, the participants’ satisfaction with the developed GART was reported to be significantly higher than “0” (moderate satisfaction) after the experiment (*t*(35) = 3.62, *p* = 0.001, *d* = 0.60). Patients with ADHD (*M* = 1.56, 95% CI [0.79, 2.32]) and HC (*M* = 0.61 (95% CI [-0.36, 1.58]) were similarly satisfied with their VR experience (*t*(34) = 1.61, *p* = 0.116, *d* = 0.55).

### Recognition task

Of the 60 potential distractors shown to participants after the experiment as a recognition test, only 50% actually represented GART-implemented distractors. Patients with ADHD (*M* = 70.46%; 95% CI [65.76%, 75.17%]) and HC (*M* = 72.71%; 95% CI [68.96%, 76.46%]) classified the presented distractors with similar recognition accuracies (*t*(32) = -0.78, *p* = 0.443, *d* = -0.27).

### Correlation analyses

**Fig. 5:**
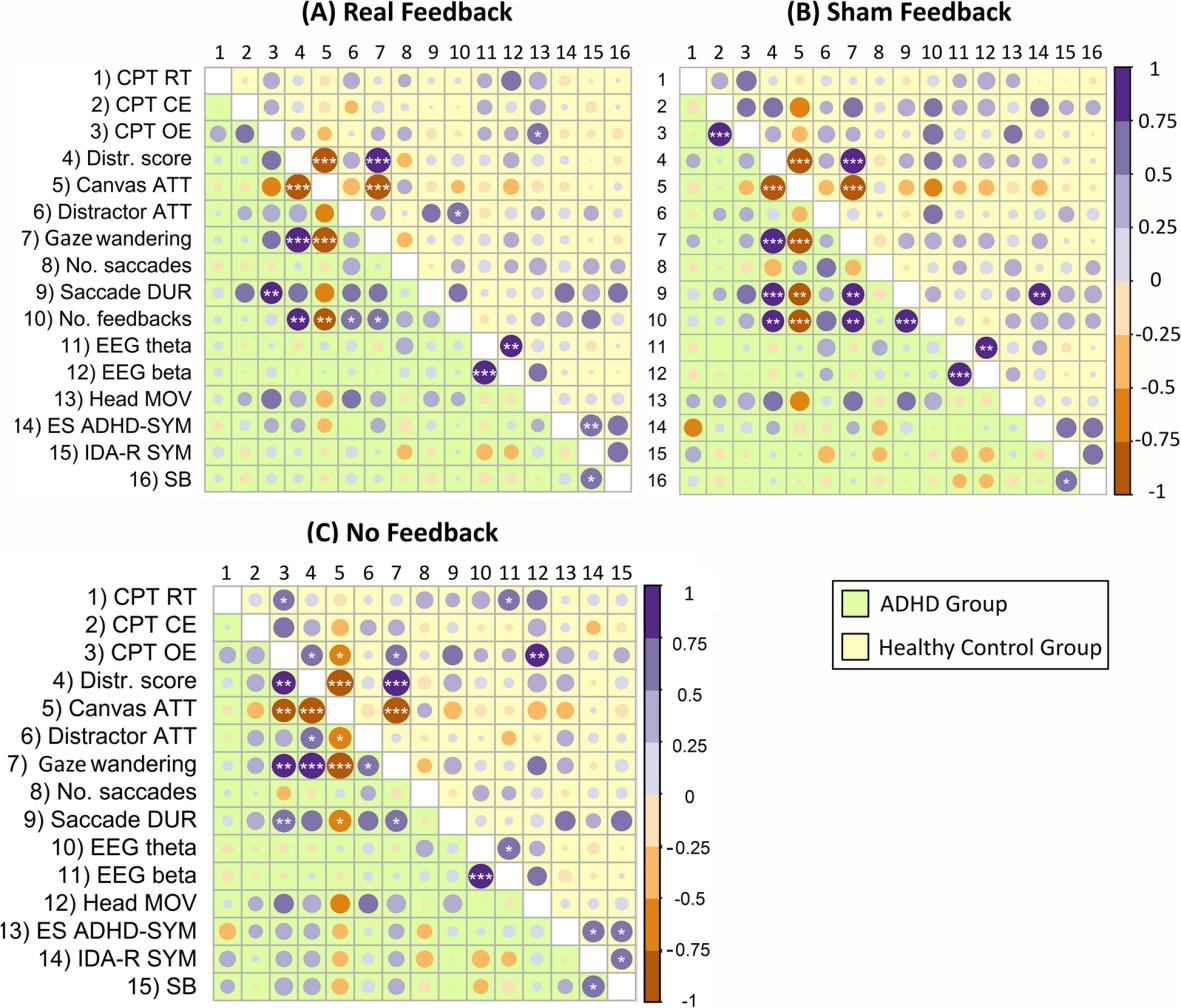
Exploratory correlation analysis. Correlation matrices including indications of statistical significance based on Benjamini–Hochberg corrected p-values are separately reported for both groups, the ADHD group (left of and below the diagonal) and HC (right of and above the diagonal). Correlations were calculated separately for the (**A**) real feedback, (**B**) sham feedback and (**C**) no feedback condition. Accordingly, correlations with the number of triggered feedback are not presented for the latter condition. The color coding of the strength of the Pearson correlations is shown on the right. Higher contrasts and greater circle seizes indicate stronger correlations. *Abbreviations*: Canvas ATT: Time of task focus indicated by attended canvas dwell times, CE: Commission errors, CPT: Continuous performance task, Distractor ATT: Attended distractors percentage dwell times, Distr. Score: Distractibility score, ES ADHD-SYM: Experience sampling self-rated ADHD symptoms, Head MOV: Head movements, IDA-R SYM: ADHD symptoms observer-rated via the IDA-R, No. feedbacks: Total number of feedback triggered, No. saccades: Total number of saccades, OE: Omission errors, RT: Reaction times, Saccade DUR: Average saccade durations, SB: ADHD symptoms self-rated via the ADHS-SB. *p < .05, **p < .01, ***p < .001

Correlation matrices for several primary and secondary outcome parameters are depicted separately for groups and feedback conditions in Fig. 5. Correlation analyses across all feedback conditions revealed clusters of strong correlations within measurement domains (e.g. between time of task focus and time of gaze wandering). In ADHD but not in HC, saccade durations appeared to correlate positively with other physiological measures of inattention, such as CPT omission errors and gaze wandering, and negatively with times of task focus under various feedback conditions. Regarding EEG, the theta and beta power were positively correlated across feedback conditions and groups. Of note, participant age and education were included as the only demographic parameters and, besides a negative correlation between education and time of distractor focus in HC during sham feedback (Spearman’s rank correlation, *r*(34) = -0.76, *p* = 0.005) and of education and number of saccades during real feedback (*r*(34) = -0.68, *p* = 0.031), no significant correlations with any parameter presented were observed.

## Discussion

In the present study, we developed a new gaze-based attention refocusing training (GART) within a virtual seminar room (VSR) in which participants automatically receive immediate feedback whenever their eye gaze behavior indicates that their visual attention has shifted away from a continuous performance task (CPT). To evaluate the general feasibility and effectiveness of the GART, 18 adult outpatients with ADHD and 18 HC performed a CPT under three different feedback conditions (real feedback, sham feedback and no feedback) and under alternating phases of high (DP) and low distraction (NDP), while they simultaneously underwent a comprehensive multimodal assessment (neuropsychological performances, eye-tracking, EEG, head actigraphy, experience sampling).

Considering the potential of VR experiments to elicit cybersickness, the here presented GART showed promising results. More specifically, all participants completed the experiment without any interruptions caused by discomfort and no adverse events were reported. Consistent with this, there was substantial satisfaction with the VR experience, particularly in the ADHD group. Overall, we observed high tolerability and feasibility of this multimodal VSR evaluation concerning the application in healthy individuals and in patients with ADHD, with overall good data quality and little data loss.

However, we did not find clear evidence of a direct effect of our training on any outcome measure. For CPT performance as the primary outcome of this study, we found comparable error rates and reaction times under our newly-developed gaze-based attention training (real feedback) and under our two implemented control conditions (sham feedback, no feedback). One reason for this might be that in this study, each feedback condition was tested only within a single CPT block of 18 min. This duration was presumably too short to effectively practice the metacognitive and refocusing strategies anticipated by our GART. As with neurofeedback, the current feedback system may also build upon learning processes that commonly involve a series of slow consolidation processes over several weeks and sessions, and which only gradually lead to improvements in cognitive performance [65].

Another unexpected finding was observed in the ADHD group, in that commission and omission errors were descriptively highest during the sham condition, followed by the real feedback condition. This might be considered to indicate additional distraction caused by the feedback stimulus itself, especially if it occurs unexpectedly, and is also reflected in the evaluation of head actigraphy, which suggests a tendency for more head movements during sham feedback. Higher levels of distraction caused by the feedback stimuli, which may even exacerbate ADHD-related symptoms, would be consistent with the present findings demonstrating an increase in omission errors and a tendency toward more commission errors during DP. Additionally, although not statistically significant and of moderate effect size, yet of potential interest for future investigations of such a gaze-based feedback procedure, the fastest reaction times were found while applying the real feedback across groups and distraction phases. This might be consistent with previous findings related to the state regulation hypothesis, according to which motivational factors, for instance, can be used to improve reaction time performance, especially in ADHD [66, 67].

In the group comparison of patients with ADHD and HC, on the other hand, we found promising evidence that our multimodal symptom assessment can discriminate well between both populations based on the findings within several measurement domains. Such a more holistic evaluation system could be of particular value in treatment outcome evaluations and the clinical assessment of ADHD, especially considering the large heterogeneity that patients with ADHD exhibit. Specifically, comparing CPT performances of the two groups across feedback conditions and distractor phases, patients with ADHD made more omission errors and reacted more slowly than HC. Previous research comparing children and healthy controls in a virtual CPT further indicated specifically increased distractor-induced performance deficits in ADHD [68]. However, while our implementation of phases of additional distraction also led to reduced CPT performances compared to phases without such additional distractors, no group interactions were observed. In the interpretation of group effects of this study, the presence of demographic differences between the groups should further be taken into consideration. The average age was higher and the average education level was lower in patients with ADHD. Older individuals, for instance, might adapt less quickly to the use of new technology compared to younger individuals. Regarding the participants’ gaze behavior, patients with ADHD spent more time gazing at presented distractors than healthy individuals. These findings relate well to previous evaluations of gaze behavior in adults with ADHD during a non-virtual CPT [69], with higher dwell times at task-irrelevant areas and distractors impacting eye movements of patients more strongly than those of HC. Notably, in the present study, patients and HC performed similarly accurate in the post-experimental recognition of distractors. This suggests that healthy individuals comparably shift their attention to distracting events, but are able to disengage their attention from those events more quickly.

The present EEG analysis revealed no group differences in the TBR. Previous reviews have provided reasonable evidence of an enhanced TBR in ADHD, although reporting age-dependence and limitations in terms of comorbidities [70]. More recent reviews, however, found smaller effect sizes in adolescents compared to children [71] and no consistent evidence for atypical TBR in adults with ADHD [72]. While this is in line with the present findings, our results should be interpreted under consideration of the higher age of the ADHD group. Notably, similar to CPT omission errors, and the number and duration of saccades, we found higher TBRs during DP than NDP, but no significant group interactions.

Head movements were identified as the only outcome parameter that distinguished ADHD from other clinical patient groups in a recent study on the combined measurement of CPT performance and head actigraphy for the differential diagnosis of ADHD in adults [73]. Our results are consistent with their findings and the general consensus in ADHD research regarding actigraphy measures [40], in that patients with ADHD initiated more head movements than HC across all feedback conditions and distraction phases.

Experience sampling, which was conducted as an in vivo time sampling of self-rated ADHD symptoms at the end of each feedback condition, revealed higher scores of inattention, hyperactivity, and impulsivity in patients with ADHD than in HC. This can be considered an important finding for future evaluations of symptoms and treatment outcome, as the assessment of symptoms in ADHD is commonly based on retrospective reports, which require sufficient metacognitive ability and accurate recognition. The present results are consistent with initial evidence that experience sampling can reflect specific ADHD symptoms in the moment [74].

Our exploratory correlation analyses revealed clusters of strong correlations within measurement domains, such as among EEG or gaze parameters. Additionally, some group-specific associations were found. For instance, only in ADHD, saccade durations were positively correlated with CPT omission errors and gaze wandering, and negatively correlated with on-canvas gaze times. Self-rated ADHD symptoms during experience sampling were more associated with retrospectively self- and observer-rated symptoms in the HC group than in the ADHD group. This possibly suggests some specificity of such in-the-moment assessments of symptoms in adult ADHD that may not be recalled in later retrospective evaluations.

This study has some limitations. First, there were demographic differences between the groups as no matching for age and education was performed, with higher age in the ADHD and a higher education level in the HC group. This may have influenced our results concerning group effects, as, for example, individuals with higher levels of education may have different abilities in processing information than individuals with lower levels of education. Yet, age did not correlate with any of the present measures, and education also did not seem to have a major impact with respect to the correlational results. Therefore, and since the implementation of covariates in smaller samples should be considered carefully, the analyses were performed as planned and as preregistered without including covariates. With respect to ethnicity, the sample is representative of the area in which the study was conducted, but its generalizability may be limited.

Second, while this study was not designed to longitudinally evaluate treatment effects of a multi-session feedback training and instead is an evaluation of the direct impact and feasibility of such a gaze-based attention feedback during a multimodal ADHD symptom assessment, indications of some additional distracting effect of the sham feedback on patients with ADHD were unexpected. This implies that we cannot rule out that confusion generated by randomized feedback stimuli in the sham condition carried over to the feedback condition. As this was a single-session experiment with only small breaks of about two minutes, no sufficient washout periods between conditions were performed. Future trials should therefore consider incorporating a patient control group that receives sham feedback and implementing a multi-session repeated measures design.

Third, while medication had to be withheld before the intervention, several patients in this sample were generally taking medication for ADHD. Consequently, possible delayed effects of ADHD medication intake, particularly on physiological measurements, need to be taken into account.

Finally, the feedback presented here uses gaze locations unimodally as an input for the feedback, while other parameters, such as periods of increased head movements, ERP components (that were left out of the present analysis due to length constraints), or specific eye movement characteristics, might be of interest for future studies as well. However, there are also technical limitations to advanced eye-tracking analysis, as VR-based eye-tracking is currently still limited to sampling rates below 300 Hz, which is, for example, considered the minimum for evaluating microsaccades [75]. Also, the feedback stimulus itself could be adapted, for instance, by providing a more ecologically valid feedback based on an avatar briefly guiding the participant, or by providing audio-only feedback that is less intrusive.

## Conclusions

We demonstrate the feasibility of gaze-based attention training using VR and multimodal assessments in adults with ADHD. However, we did not find a direct effect of gaze-based feedback on attentional performance. There were indications that sham feedback elicited particularly negative responses in patients with ADHD. We propose future longitudinal, multi-session trials to determine the prerequisites for potential initiations of learning processes similar to neurofeedback procedures to derive a therapeutic potential for adult ADHD. The differentiation of patients with ADHD from healthy individuals yielded promising results in this virtual seminar room study: patients made more omission errors and showed higher CPT reaction times, had higher distractor-related dwell times, moved their heads more, and self-reported higher ADHD symptoms during task engagement. A more holistic, multimodal assessment, such as the one proposed here, might adequately grasp the heterogeneity of ADHD symptomatology and potentially provide an exploratory set of biomarkers, thereby taking another step toward precision medicine in ADHD.

## Supplementary Information

**Additional file 1.** A short video presentation of the virtual scenario in first person perspective. A user performs the continuous performance task in the developed VSR. Two exemplary distractors are presented and audiovisual feedback is played based on the gaze behavior.

**Additional file 2. **Detailed results of all conducted ANOVA procedures (Supplementary Tables 1 – 5).

## Data Availability

The dataset and analysis code supporting the conclusions of this article are available in the Open Science Framework (OSF) repository, https://osf.io/6a23b (https://doi.org/10.17605/OSF.IO/6A23B).

## Abbreviations

ADHD: Attention-deficit/hyperactivity disorder
CPT: Continuous performance task
CCT: Computerized cognitive training
VR: Virtual reality
TBR: Theta/beta ratio
GART: Gaze-based attention refocusing training in virtual reality
VSR: Virtual seminar room
HC: Healthy control
DP: Distractor phases
NDP: Non-distractor phases
IDA-R: Integrated Diagnosis of ADHD in Adulthood
MINI-DIPS: Brief Diagnostic Interview for Mental Disorders
ADP-IV: Assessment of DSM-IV Personality Disorders
ADHS-SB: Self-rating behavior questionnaire
WHOQOL: World Health Organization Quality Of Life questionnaire
DASS: Depression, Anxiety and Stress Scales
HMD: Head-mounted display
VRSQ: Virtual Reality Sickness Questionnaire
LSL: Lab Streaming Layer
ICA: Independent component analysis
