# Supplementary material for: Gaze-based attention refocusing training in virtual reality for adult attention-deficit/hyperactivity disorder
Source: BMC Psychiatry. 2023 Jan 26;23:74. doi: 10.1186/s12888-023-04551-z (PMC9879564; doi:10.1186/s12888-023-04551-z)
Supplement: Supplementary file 2 — Additional file 2. Detailed results of all conducted ANOVA procedures (Supplementary Tables 1 – 5). [file 12888_2023_4551_MOESM2_ESM.pdf]

Supplementary Material 2

Gaze-based Attention Refocusing Training in Virtual Reality for Adult Attention-Deficit/Hyperactivity Disorder

Benjamin Selaskowski, Laura Marie Asché, Annika Wiebe, Kyra Kannen, Behrem Aslan, Thiago Morano Gerding, Dario Sanchez, Ulrich Ettinger, Markus Kölle, Silke Lux, Alexandra Philipsen & Niclas Braun

Supplementary Table 1: Results of mixed 2 × 3 × 2 ANOVAs on CPT parameters

Supplementary Table 2: Results of Group × Block mixed ANOVAs (2 × 3) on gaze parameters and Group × Block × Phase mixed ANOVAs (2 × 3 × 2) on saccade parameters

Supplementary Table 3: Results of mixed 2 × 3 × 2 ANOVAs on EEG parameters

Supplementary Table 4: Results of mixed 2 × 3 × 2 ANOVA on actigraphy parameter

Supplementary Table 5: Results of mixed 2 × 3 ANOVAs on experience sampling parameters

Supplementary Table 1. Results of separate Group × Block × Phase mixed ANOVAs (2 × 3 × 2) on Commission Errors, Omission Errors and Reaction Times of the CPT.

Condition	df	F	*p*	η_p^2
Commission Errors				
Block (GG)	1.04, 35.43	1.068	.312	.030
Block × Group (GG)	1.04, 35.43	0.835	.372	.024
Phase	1, 34	3.741	.061	.099
Phase × Group	1, 34	0.443	.510	.013
Block × Phase	2, 68	0.214	.808	.006
Block × Phase × Group	2, 68	1.088	.343	.031
Group	1, 34	3.057	.089	.082
Omission Errors				
Block (GG)	1.28, 43.42	0.466	.545	.014
Block × Group (GG)	1.28, 43.42	1.197	.293	.034
Phase	1, 34	9.350	.004	.216
Phase × Group	1, 34	3.447	.072	.092
Block × Phase (HF)	1.85, 62.82	0.338	.697	.010
Block × Phase × Group (HF)	1.85, 62.82	0.394	.659	.011
Group	1, 34	5.565	.024	.141
Reaction times				
Block	2, 68	2.616	.080	.071
Block × Group	2, 68	0.018	.982	.001
Phase	1, 34	0.422	.520	.012
Phase × Group	1, 34	2.258	.142	.062
Block × Phase	2, 68	0.100	.905	.003
Block × Phase × Group	2, 68	0.130	.878	.004
Group	1, 34	4.372	.044	.114

Note. CPT: Continuous Performance Task, GG: Greenhouse-Geisser adjusted, HF: Huynh-Feldt adjusted.

Supplementary Table 2. Results of separate Group × Block mixed ANOVAs (2 × 3) on Gaze Parameters and Group × Block × Phase mixed ANOVAs (2 × 3 × 2) on Saccade Parameters.

Condition	df	F	*p*	η_p^2
Distractibility score				
Block (HF)	1.71, 58.17	0.193	.791	.006
Block × Group (HF)	1.71, 58.17	0.228	.763	.007
Group	1, 34	3.676	.064	.098
Relative canvas gaze time				
Block	2, 68	0.107	.899	.003
Block × Group	2, 68	0.655	.523	.019
Group	1, 34	3.483	.071	.093
Relative distractors gaze time				
Block (HF)	1.83, 62.04	0.734	.472	.021
Block × Group (HF)	1.83, 62.04	0.574	.551	.017
Group	1, 34	9.404	.004	.217
Relative gaze wandering time				
Block	2, 68	0.195	.823	.006
Block × Group	2, 68	0.540	.585	.016
Group	1, 34	2.577	.118	.070
Average saccade durations				
Block	2, 68	1.439	.244	.041
Block × Group	2, 68	1.503	.230	.042
Phase	1, 34	11.733	.002	.257
Phase × Group	1, 34	0.551	.463	.016
Block × Phase (HF)	1.84, 62.69	0.881	.412	.025
Block × Phase × Group (HF)	1.84, 62.69	0.386	.665	.011
Group	1, 34	3.897	.057	.103
Number of saccades				
Block	2, 68	1.919	.155	.053
Block × Group	2, 68	0.159	.853	.005
Phase	1, 34	13.870	.001	.290
Phase × Group	1, 34	2.085	.158	.058
Block × Phase	2, 68	0.023	.977	.001
Block × Phase × Group	2, 68	0.903	.410	.026
Group	1, 34	4.897	.034	.126

Note. HF: Huynh-Feldt adjusted.

Supplementary Table 3. Results of separate Group × Block × Phase mixed ANOVAs (2 × 3 × 2) on the EEG Theta/Beta Ratio.

Condition	df	F	*p*	η_p^2
Theta/Beta ratio				
Block	2, 68	0.752	.475	.022
Block × Group	2, 68	2.071	.134	.057
Phase	1, 34	18.015	< .001	.346
Phase × Group	1, 34	3.408	.074	.091
Block × Phase	2, 68	0.131	.877	.004
Block × Phase × Group	2, 68	0.463	.632	.013
Group	1, 34	0.253	.618	.007

Supplementary Table 4. Results of the Group × Block × Phase mixed ANOVA (2 × 3 × 2) on Head Movements.

Condition	df	F	p	η_p^2
Head movements				
Block	2, 68	3.582	.033	.095
Block × Group	2, 68	1.449	.242	.041
Phase (HF)	1, 34	0.039	.845	.001
Phase × Group (HF)	1, 34	0.738	.396	.021
Block × Phase (HF)	1.82, 61.79	0.101	.887	.003
Block × Phase × Group (HF)	1.82, 61.79	0.540	.569	.016
Group	1, 34	16.060	< .001	.321

Note. HF: Huynh-Feldt adjusted.

Supplementary Table 5. Results of separate Group × Block mixed ANOVAs (2 × 3) on Parameters of Experience Sampling.

Condition	df	F	p	η_p^2
Inattention				
Block	2, 68	0.464	.631	.013
Block × Group	2, 68	0.250	.780	.007
Group	1, 34	19.574	< .001	.365
Hyperactivity				
Block	2, 68	0.359	.700	.010
Block × Group	2, 68	1.053	.354	.030
Group	1, 34	16.958	< .001	.333
Impulsivity				
Block	2, 68	0.742	.480	.021
Block × Group	2, 68	0.673	.513	.019
Group	1, 34	8.764	.006	.205
